# Liposome Sterile Filtration Characterization via X-ray Computed Tomography and Confocal Microscopy

**DOI:** 10.3390/membranes11110905

**Published:** 2021-11-22

**Authors:** Thomas F. Johnson, Kyle Jones, Francesco Iacoviello, Stephen Turner, Nigel B. Jackson, Kalliopi Zourna, John H. Welsh, Paul R. Shearing, Mike Hoare, Daniel G. Bracewell

**Affiliations:** 1 Department of Biochemical Engineering, University College London, Bernard Katz, London WC1E 6BT, UK; thomas.johnson.10@ucl.ac.uk (T.F.J.); m.hoare@ucl.ac.uk (M.H.); 2 Pall Corporation 5 Harbourgate Business Park, Southampton Road, Portsmouth PO6 4BQ, UK; kyle_jones@europe.pall.com (K.J.); stephen_turner@europe.pall.com (S.T.); nigel_jackson@europe.pall.com (N.B.J.); kalliopi_zourna@europe.pall.com (K.Z.); john_welsh@europe.pall.com (J.H.W.); 3 Electrochemical Innovation Laboratory, Department of Chemical Engineering, University College London, Torrington Place, London WC1E 7JE, UK; f.iacoviello@ucl.ac.uk (F.I.); p.shearing@ucl.ac.uk (P.R.S.)

**Keywords:** sterile filtration, liposomes, X-ray computed tomography, confocal microscopy

## Abstract

Two high resolution, 3D imaging techniques were applied to visualize and characterize sterilizing grade dual-layer filtration of liposomes, enabling membrane structure to be related with function and performance. Two polyethersulfone membranes with nominal retention ratings of 650 nm and 200 nm were used to filter liposomes of an average diameter of 143 nm and a polydispersity index of 0.1. Operating conditions including differential pressure were evaluated. X-ray computed tomography at a pixel size of 63 nm was capable of resolving the internal geometry of each membrane. The respective asymmetry and symmetry of the upstream and downstream membranes could be measured, with pore network modeling used to identify pore sizes as a function of distance through the imaged volume. Reconstructed 3D digital datasets were the basis of tortuous flow simulation through each porous structure. Confocal microscopy visualized liposome retention within each membrane using fluorescent dyes, with bacterial challenges also performed. It was found that increasing pressure drop from 0.07 MPa to 0.21 MPa resulted in differing fluorescent retention profiles in the upstream membrane. These results highlighted the capability for complementary imaging approaches to deepen understanding of liposome sterilizing grade filtration.

## 1. Introduction

Liposomes consist of a lipid sphere that can carry a payload either within the bilayer or the internal cavity [1,2,3] which is being increasingly explored for advanced therapies [4,5,6]. The first liposome-based products for therapeutic purposes were licensed in the 1990s [7,8,9] and have expanded into multiple applications since then, from anti-cancer treatments [10,11,12] to vaccines for COVID-19 [13,14,15]. The advantages of using liposomes include improved drug solubility and absorption in addition to potential dosage reductions [4,16]. Liposomes are expected to become increasingly popular as delivery vehicles for the next generation of medicines, including gene therapies [17].

Sterile filtration is a ubiquitous unit operation in biological medicines manufacturing and is typically performed at the end of a bioprocess [18,19,20]. Liposomes present several challenges at the sterile filtration stage when compared to conventional biologics such as monoclonal antibodies [21,22,23], with one of the most significant being that liposomes are much larger and closer in size to the nominal retention rating (NRR) of a sterile membrane [24,25,26,27]. This larger size may result in increased risk of product loss, membrane fouling and bacterial penetration that would impact processing efficiency through blocking mechanisms that are not prevalent when filtering antibodies [28,29].

The need to manufacture high-quality medicines at scale requires suitable and effective manufacturing processes. The bioprocessing challenges presented during sterilizing grade filtration of liposomes require a greater fundamental understanding of fouling so that the operating conditions, lipid envelope properties and media selection can be optimized.

Sterilizing grade membranes have an internal, 3D structure that is essential to function and performance. A nominal retention rating, which can be considered as a size threshold that the filter will effectively retain, of 200 nm is standard for sterilizing grade filters [30]. By having a submicron pore size, a membrane may be susceptible to fouling and so an upstream layer with a larger nominal retention rating may be included to reduce flux decay or pressure increases depending on the mode of operation [31]. Membrane internal structure can either be symmetric or asymmetric, with changes to pore size and the resultant alterations to flow path tortuosity and performance of particular interest.

High resolution imaging techniques have advanced to the point where they are capable of visualizing and characterizing the 3D structure of a material at the submicron scale for a wide range of applications [32,33,34]. Individual imaging techniques often have advantages and disadvantages and so should be selected in relation to the sample of interest [35]. For example, serial block face microtomy and focused ion beam microscopy can achieve nanoscale resolutions, however, require extensive preparation of a sample which is destroyed during the imaging process [36,37,38]. By imaging the internal geometry and feed material fouling within sterilizing grade filters then membrane structure can be better related to function and performance for lipid enveloped products.

Characterization of pore size distribution, tortuosity and permeability are of particular interest and importance in understanding the internal structure of materials. Accurately representing these properties requires a sufficient imaging pixel size in order to view the finest features and sub-porous structure within a sample; however, this can result in compromises to other factors including imaging field of view.

X-ray computed tomography (CT) is a 3D imaging technique that can achieve submicron resolutions whilst reducing sample preparation and without destroying the sample during image acquisition [39]. X-ray CT has been previously used to image and evaluate the submicron structure of chromatography beads used in bioprocessing [40]; however, the technique was not deemed suitable for imaging separation materials fouled with biological materials [41].

In order to image both the internal structure of membranes and the location that liposomes and bacteria are retained a complementary imaging technique to X-ray CT is required. Confocal Laser Scanning Microscopy (CLSM) was determined to be best suited for imaging feed material entrapment within membranes [42,43,44,45]. CLSM can measure fluorescent dyes in 3D space within materials.

By labelling a species of interest with such a dye, in this study liposomes and bacteria, they can be located within the membrane structure. By combining tomographic data from X-ray CT and CLSM, structural measurements and internal retention properties of sterilizing grade membranes can be analyzed. Using this methodology, the impact of membrane nominal retention rating, asymmetry and operating conditions, including differential pressure, are investigated here to develop a greater understanding of fouling during sterile filtration of liposomes.

## 2. Materials and Methods

### 2.1. Membranes and Filtration

Two polyethersulfone (PES) membranes (Pall Corporation, Portsmouth, UK) were examined in this study: an asymmetric 650 nm rated upstream layer and a symmetric 200 nm rated downstream layer. Both membranes were housed in a Pall 47 mm stainless steel disc holder with tests run at constant differential pressures between 0.07 and 0.21 MPa at room temperature. Direct flow filtration was performed using a compressed air supply and pressure vessel.

A M110EH Microfluidizer^®^ (Microfluidics Corporation, Newton, MA, USA) was used to produce liposomes that were composed of Lipoid S 100 (1.5%wt) and soybean oil (5%wt) which were mixed with pure water before operation. The first two passes through the microfluidizer were performed at 140 MPa and a third pass at 210 MPa. For confocal studies, Topfluor^®^ PC (Avanti Polar Lipids Inc., Alabaster, AL, USA) was included in solution at a 0.05% concentration.

Liposome size and zeta potential were measured using Dynamic Light Scattering (DLS), where samples were diluted in Phosphate Buffered Saline (PBS) (Sigma-Aldrich, Gillingham, UK) before being pipetted into cuvettes and analyzed using a Zetasizer Nano ZS (Malvern, Malvern, UK). Brevundimonas diminuta (B. dim) solution was provided by Pall Corporation for bacterial challenges at a concentration of 3 × 10^7^ CFU⋅mL^−1^. BacLight™ stain (Thermo Fisher Scientific, Loughborough, UK) was used to dye the bacterial solutions for confocal studies. Following filtration of dyed liposomes each individual membrane was removed for imaging in separate confocal microscopy acquisition sessions.

### 2.2. D Imaging and Analysis

CLSM was performed on membranes that had undergone dyed liposome filtration and bacterial challenge using a TCS SP8 Upright Microscope (Leica Microsystems, Germany) with a 63x oil immersion lens. Accompanying Leica LAS X software was used to control scanning of each membrane sample to capture images at 346 nm intervals. Images were loaded into ImageJ and aligned using the StackReg plugin. The average intensity of liposome and B. dim fluorescence was measured as a function of distance through the membrane sample.

**Figure 1:**
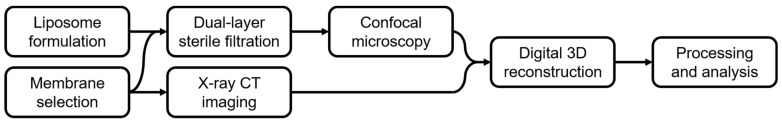
Experimental flow schematic of filtration and imaging experiments.

Nanoscale X-ray CT was performed using an Xradia 810 Ultra (Zeiss, Pleasanton, CA, USA) achieving a pixel size of 63 nm. Membrane samples were cut into 1 mm squares before being mounted and adhered to the top of a pinhead in preparation for imaging. In each case, 1601 projections were acquired over a 180° sample rotation. Figure 1 displays the overall process for liposome imaging and high resolution imaging used in this study.

Reconstruction of datasets into 3D TXM volume files was performed using Reconstructor XM (Zeiss, Pleasanton, CA, USA) software which were loaded into Avizo^®^ Fire 9.5 (Thermo Fisher Scientific, Waltham, MA, USA) for further processing and analysis. Commands including ‘Unharp masking,’ ‘Non-local means’ and ‘Interactive thresholding’ were used to eliminate artefacts and segment digital samples into material and voidage phases. The voidage phase was visualized using the ‘Chamfer Distance Map’ and ‘Separate Objects’ was applied before ‘Pore Network Modeling’ that allowed for pore size and connectivity to be viewed, in addition to generation of pore size distributions. The XLAB plugin was applied for permeability simulation on each subvolume using a differential pressure of 0.1 MPa and a fluid viscosity of 8.9 × 10^−4^ Pa⋅s. The material phase was exported as 3D TIFF files into the MatLab^®^ plugin TauFactor [46] for tortuosity evaluation [47,48,49,50].

## 3. Results and Discussion

### 3.1. Membrane Structure Imaging

X-ray CT was selected for imaging the internal structure of both PES membranes due to the resolutions achievable being suitable for visualizing fine porous features. X-ray CT is non-destructive, hence allowing for multiple acquisition sessions to optimize imaging settings. Alternative techniques with comparable resolutions including focused ion beam microscopy require more extensive sample preparation when compared with X-ray CT and so were not explored in this study. A small cut-out of membrane was required for nanoscale X-ray CT imaging. Resolution and field of view constraints necessitated internal tomography and multiple imaging sessions to reconstruct and visualize membrane porous structure.

**Figure 2:**
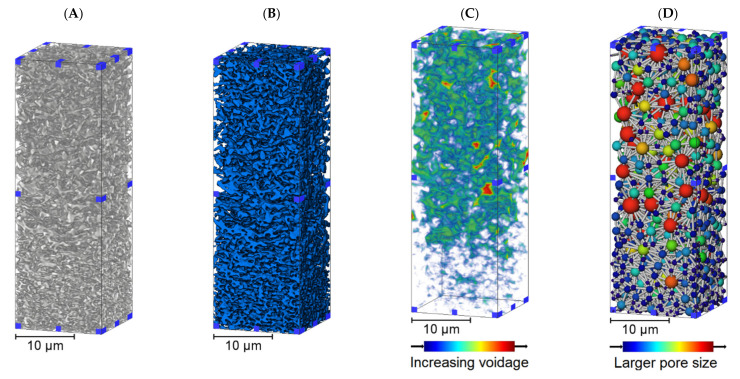
The 3D subvolume renders of the upstream membrane from X-ray CT imaging. Digital representations of the same sample are displayed following reconstruction, processing and phase segmentation. (**A**) A 3D render of an imaged subvolume. (**B**) A 3D render of the subvolume following segmentation with blue representing the material phase. (**C**) Voidage distance map displaying the largest spaces within the subvolume. (**D**) Pore Network Modeling within the subvolume.

The two samples examined in this study were an upstream asymmetric membrane followed by a downstream symmetric membrane. The respective NRRs were 650 nm and 200 nm, with 200 nm being the standard rating implemented for sterile filtration. Asymmetry in the upstream membrane allows for larger debris and particles to be collected in the support structure, reducing the burden on the downstream membrane that would be more susceptible to fouling without the upstream counterpart. Figure 2 displays 3D renders of a subvolume reconstructed from the upstream membrane, including a voidage distance map and a pore network. These subvolume renders are the same upstream membrane sample image acquisition and so represent the material and voidage phases.

**Figure 3:**
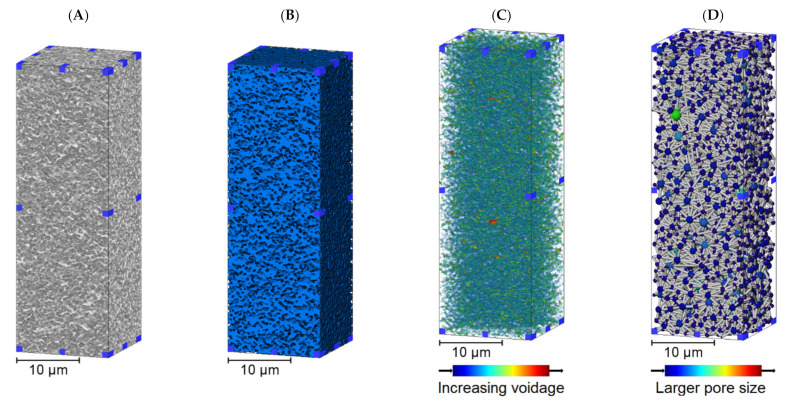
The 3D subvolume renders of the downstream membrane from X-ray CT imaging. (**A**) A 3D render of an imaged subvolume. (**B**) A 3D render of the subvolume following segmentation with blue representing the material phase. (**C**) Voidage distance map displaying the largest spaces within the subvolume. (**D**) Pore Network Modeling within the subvolume.

The 3D renders display the asymmetry within the upstream membrane subvolume, where the structure is more open in the upper third before transitioning to a tighter structure in the lower third. Both voidage distance map and pore models visually suggest asymmetry with increasing voidage and larger pore sizes being observed in the upper third of the subvolume. The upstream membrane is a single sheet where the internal structure changes as a function of distance through the membrane. Filter structure is integral to both function and performance, with larger voids upstream enabling debris carrying capacity that could foul the downstream membrane; corresponding representations from X-ray CT are available in Figure 3.

**Figure 4:**
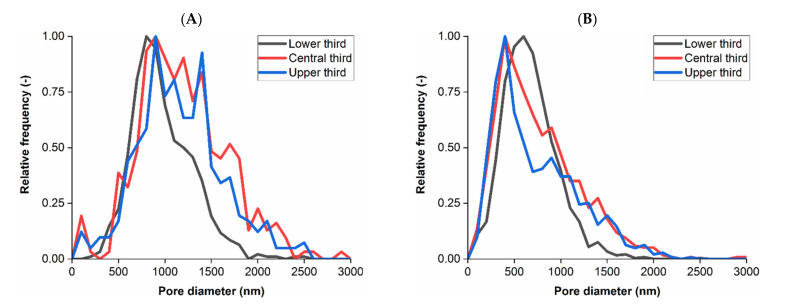
Membrane pore size distribution. (**A**) Upstream membrane subvolume, 650 nm nominal retention rating. Average pore size for each third: upper 1115 nm, central 1149 nm, lower 938 nm. (**B**) Downstream membrane subvolume, 200 nm nominal retention rating. Average pore size for each third: upper 616 nm, central 711 nm, lower 686 nm. Subvolumes were split into thirds for positional based analysis.

The downstream membrane is a symmetric material and therefore no obvious changes to structure through the subvolume were expected. The 3D renders in Figure 3 do suggest a relatively consistent structure, voidage and pore network in comparison to the upstream membrane although there are instances of larger voids and pores in places. The visualizations of each membrane structure and voidage properties indicated that there were differences between the upstream and downstream layers. The pore size distributions resulting from Pore Network Modeling of segmented volumes within each membrane are displayed in Figure 4 as a function of upper, middle and lower third of each sample.

The average pore diameter based on a 63 nm pixel size for the upstream membrane subvolume was 1032 nm ± 414 nm (1 standard deviation) and 661 nm ± 378 nm for the downstream subvolume, whereby fewer than 17% of pores were larger than 1000 nm in the downstream subvolume. For both membranes the average pore size from pore network modeling was greater than the respective NRR.

Improving pixel size further may enable finer pores to be resolved or better defined, which may have reduced the average pore size further for both membranes, in particular the downstream subvolume. However, improving pixel size would have resulted in a considerable field of view compromise that would have made imaging a sufficient volume for analysis difficult. The pore size distribution of the upstream membrane lower third was smaller compared to the other volumes, whilst the downstream membrane subvolumes were more consistent although did display a degree of variability. The pore size distributions in the upper and central thirds of the upstream membrane appeared similar rather than displaying a gradual transition between larger pores to tighter structure in the lower third, indicating that the tightening of membrane geometry may occur over a short distance. Improving imaging resolution, which may include using alternative techniques previously described in the Introduction, may be capable of characterizing this transition by imaging the finest pores, however would require compromises to field of view capabilities and also may necessitate further sample preparation.

Reconstructed volumes from X-ray CT were evaluated for permeability using the XLAB plugin for Avizo^®^ and tortuosity using the TauFactor [46] plugin for MatLab^®^ to compare between membrane subvolumes and individual thirds. Membrane permeability is important in governing characteristics including pressure increase or flux decrease during filtration, with operating conditions key to determining both yield and throughput. One aspect of this, tortuosity, can be considered as the effective path length through a porous material, which has historically been derived through equation-based derivations but may now be directly evaluated or simulated using 3D data and appropriate software [50]. Results from permeability and tortuosity simulations are available in Table 1.

There were two main observations from Table 1, the first being that permeability decreased and tortuosity factor increased from upper to lower third in the upstream membrane (ANOVA *p* < 0.05), but was consistent in the downstream membrane (*p* > 0.05). This was expected due to the visualization and pore analysis results from Figure 2, Figure 3 and Figure 4 suggesting that the internal structure of the upstream membrane was asymmetric and the downstream membrane symmetric as a function of distance through the subvolume. Quantifying symmetry in the downstream membrane confirmed that feed material will have to traverse through this tighter, more consistent membrane when compared to the upstream counterpart that will enable any remaining bacteria to be removed.

The second observation was that permeability decreased and tortuosity factor increased between the upstream and downstream subvolume (*p* < 0.05) which was expected due to the pore sizes being smaller. Using 3D data enables the intricate geometry of a real sample to be considered when evaluating tortuosity in comparison to equation-based derivations that are reliant on porosity values, for example a complex structure may not have the same tortuosity in each dimension but would have an identical overall porosity.

### 3.2. Liposome Retention Imaging

X-ray CT was used to measure internal membrane geometry; however, the technique has previously been found to be ineffective at imaging materials fouled with biological samples [41]. Confocal microscopy was selected as a complementary 3D imaging technique for imaging feed material entrapment within each membrane. Liposomes and bacteria were labelled prior to filtration and confocal microscopy imaging for fluorescence-based identification within each membrane.

**Figure 5:**
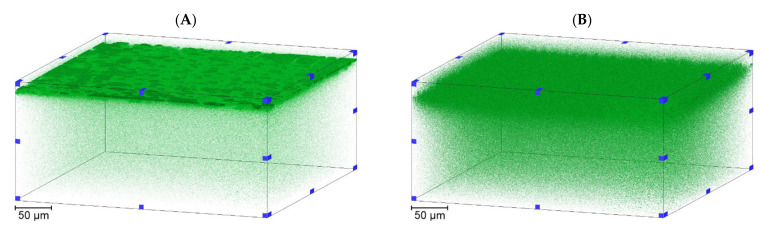
Confocal microscopy imaging of membranes following dyed liposome filtration. (**A**) A 3D render of the upstream membrane. (**B**) A 3D render of the downstream membrane. Filtration was performed at a constant differential pressure of 0.14 MPa.

Following sterilizing grade filtration, a series of 2D images from confocal microscopy were acquired that were reconstructed into a 3D volume. Producing stacks of hundreds of 2D images enabled fluorescence visualization and measurement through the entire membrane thickness. Figure 5 displays 3D fluorescence renders from filtration of liposomes without bacteria for both membranes.

Confocal microscopy was successful in identifying liposomes that were retained within each membrane, with filtration transmission exceeding 90%. The majority of retained liposome occurred in the upper third of each sample, however individual liposomes could not be sufficiently resolved for counting. The 3D reconstructions demonstrated that the internal structure was retaining liposomes across a consistent band, however there were instances of higher fluorescence that indicated some heterogeneity within the internal pore structure leading to differing levels of fouling at the intensity peak 11 µm into the membrane.

As seen in Figure 2 there is a degree of structural heterogeneity that is greatest in the more open section of the upstream membrane that may correspond to the fluorescence profiles having individual areas that are brighter due to a greater concentration of liposome retention. This could occur to due to pore morphology in a localized region causing plugging or local liposome polarization and could potentially favor adsorptive effects for liposomes compared to other flow paths in the membrane.

**Figure 6:**
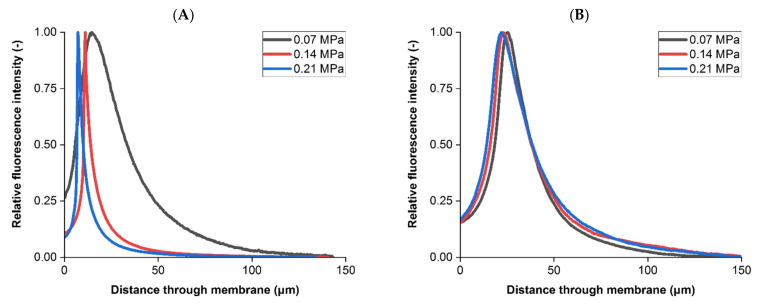
Intensity profiles of fluorescent liposome retention. (**A**) Upstream membrane profiles at three differential pressures. (**B**) Downstream membrane profiles at three differential pressures. Intensity profiles presented are based on measurements from all 2D slices from a single sample imaged using CLSM, with intensity relative to each respective peak value displayed and further compared in the text. Following dual-layer filtration each individual membrane was removed from the filter housing and imaged using confocal microscopy separately.

Jackson et al. [42] and Dishari et al. [43] previously examined bioprocessing membrane retention using confocal microscopy, focusing on viral clearance rather than sterilizing grade filtration. In both of these studies the 2D confocal images also suggest a similar retention profile within these membranes to those seen in Figure 5 and also clearly display a degree of variability and heterogeneity within the retention bands, with Dishari et al. [43] using a cross-sectional SEM to visualize internal membrane 2D structure. Effective filtration also relies on optimization of operating conditions applied to the specific feed stream and membranes being used. Three constant differential pressures were applied during liposome filtration tests, with resulting intensity profiles for both membranes compared in Figure 6.

The intensity profiles were found to be different in the upstream membrane (ANOVA *p* < 0.05, >400 slices per sample) but similar in the downstream membrane (*p* > 0.05). In the upstream membrane, increasing differential pressure resulted in a peak fluorescence closer to the sample entrance. Additionally, the peaks sharpened at increasing differential pressures, with the 0.21 MPa test recording a peak florescence intensity 1.6 times greater than at 0.14 MPa and 5.6 times greater than at 0.07 MPa. Downstream counterparts varied in peak fluorescence by 1.3 times or less with the peak downstream fluorescence values being between the lower and middle differential pressures in the upstream membrane.

Zourna et al. [23] found that across multiple dual layer PES sterilizing grade membranes, including those examined here, increasing differential pressure from 0.07 MPa to 0.21 MPa improved liposome transmission from 85–90% to near 100% and increased throughput between 5.7 to 7.4 times. Therefore, increasing differential pressure enhances conventional performance metrics for these membranes when filtering liposomes but also impacts the retention profile observed in the asymmetric, upstream membrane. As discussed by Zourna et al. [23], increasing differential pressure to drastically improve product transmission and throughput is not typical for the majority of particulate systems during filtration. Given that the downstream membrane is responsible for final bacterial removal then it is important that the structure does not easily foul and so the upstream membrane needs to be robust enough to guard against multiple operating conditions and feed stream compositions.

In that study the two mechanisms to explain improved liposome transmission and throughput at increased differential pressures were shear susceptibility of the liposomes and residence times within the membrane enabling absorption to internal surfaces. Any liposome material that successfully transmitted through the dual layer membrane system would not have been imaged using post-filtration confocal microscopy. The profiles in Figure 6 suggest that retention differences are occurring in the upstream membrane. The lowest differential pressure investigated here at 0.07 MPa produced the broadest peak in the upstream membrane which may be attributed to a greater residence time and therefore greater susceptibility for absorptive effects as suggested by Zourna et al. [23].

### 3.3. Bacterial Challenges

**Figure 7:**
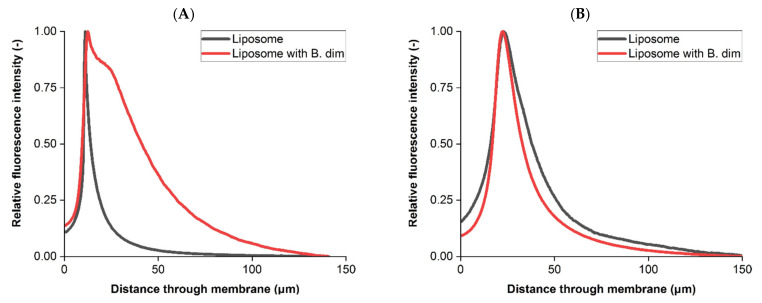
Intensity profiles of liposome retention when filtered with B. dim. (**A**) Upstream membrane profiles. (**B**) Downstream membrane profiles. Intensity profiles presented are based on measurements from all 2D slices from a single sample imaged using CLSM. Filtration was performed at a constant differential pressure of 0.14 MPa.

The purpose of sterile filtration is to reproducibly remove microorganisms from the bioprocess stream and so liposome feeds were mixed with bacteria prior to filtering. B. dim is the standard bacteria used for bacterial challenges because of a relatively small size. Folmsbee [30] discussed the possibility of potential bacterial penetration during sterile filtration of liposome products when applying greater differential pressures, therefore whilst transmission and throughput can be improved it may also risk compromising the overall process step integrity. Dyed liposomes were mixed with B. dim bacteria for filtration to compare the intensity profile with a bacteria-free liposome feed under identical operating conditions at a differential pressure of 0.14 MPa, available in Figure 7.

The relative liposome intensity profiles obtained for both membranes were found to be different (*p* < 0.05) when comparing the liposome and bacterial mix feed streams. This indicates that bacteria can influence the retention and fouling characteristics of liposome products during sterile filtration. In the upstream sample, the bacterial mix resulted in a more pronounced tail following the intensity peak when compared to the unmixed liposome feed.

Interaction between liposomes and B. dim impacted the retention profile in the downstream membrane. Whilst the peak intensity was recorded approximately 22 µm through the downstream sample for both feeds, the liposome and bacteria mix produced a broader and more sustained peak.

**Figure 8:**
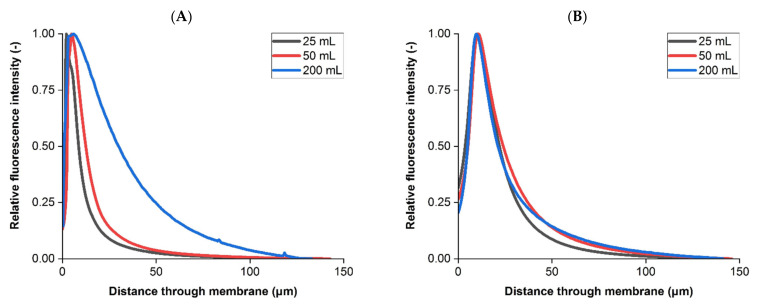
Fluorescently labelled bacterial challenges at increasing load volumes from 25 mL to 200 mL. (**A**) Upstream membrane. (**B**) Downstream membrane. Intensity profiles presented are based on averaged measurements from all 2D slices from a single sample imaged using CLSM. Filtration was performed at a constant differential pressure of 0.14 MPa.

Retention profiles for B. dim were also examined during filtration, with comparisons between load volumes of interest performed due to concerns of breakthrough when passing increasing amounts of liposome and bacteria through a dual layer sterilizing grade filter. B. dim were dyed before testing, with each membrane imaged using confocal microscopy after separate filtrations at increasing load volumes. Figure 8 displays the retention profiles of bacteria for both membranes after loading volumes between 25 to 200 mL.

All labelled bacteria filtration results in the upstream membrane presented peaks within the first 6 µm of the sample. However, each had different profiles (*p* < 0.05), with increasing load volumes from 25 mL to 200 mL producing a fluorescence peak three times less intense with a greater tail. Intensity profiles in the downstream membrane were more similar (*p* > 0.05). No bacterial breakthrough or change to the downstream retention profile was observed at the load volumes and operating conditions investigated in this study, which suggests the dual layer membrane was performing as required under the parameters investigated.

## 4. Conclusions

Liposomes and lipid enveloped products are becoming increasingly prevalent for drug delivery and vaccines, highlighting the need for efficient manufacturing at a scale that requires effective sterilizing grade filtration. Liposomes present several challenges at this stage such as their relatively large size, leading to potential processing issues that are not as pronounced for other major medicines such as monoclonal antibodies. By applying two imaging techniques in this study both the internal structure and the location of feed material retention within each membrane could be identified through positional based analysis. This approach can provide greater understanding as to where feed materials may be retained within complex geometrical structures such as the membranes studied here across multiple operating conditions and the mechanisms to which bacterial co-penetration may occur.

The two imaging techniques were selected due to their complementary capabilities. X-ray computed tomography achieved resolutions sufficient to visualize internal porous geometry of both membranes with minimal sample preparation and therefore a low potential for damage or alteration. Confocal microscopy was directly suited to imaging feed material and so was ideal for combining datasets to relate liposome and bacterial retention to 3D structural characteristics. Including further imaging and non-imaging techniques would allow for a greater understanding in relating structure to function and performance. Investigating an expanded range of operating conditions, membrane types and feed compositions for life cycle and fouling studies would be of interest for future studies.

## Figures and Tables

**Table 1:** Simulated permeability and tortuosity values. Permeability was determined using Avizo XLab and Tortuosity Factor using the Taufactor plugin for Matlab. Results are reported to three significant figures and one standard deviation.

Membrane	Location	Permeability (µm^2^)	Tortuosity Factor
**Upstream**	Upper third	0.0279 ± 0.0015	1.21 ± 0.02
	Central third	0.0257 ± 0.0013	1.24 ± 0.02
	Lower third	0.0139 ± 0.0011	1.32 ± 0.05
**Downstream**	Upper third	0.0013 ± 0.0004	2.47 ± 0.58
	Central third	0.0013 ± 0.0004	2.47 ± 0.59
	Lower third	0.0013 ± 0.0004	2.51 ± 0.61

## Data Availability

Data available on request.
